# Capivasertib combines with docetaxel to enhance anti-tumour activity through inhibition of AKT-mediated survival mechanisms in prostate cancer

**DOI:** 10.1038/s41416-024-02614-w

**Published:** 2024-02-23

**Authors:** Cath Eberlein, Stuart C. Williamson, Lorna Hopcroft, Susana Ros, Jennifer I. Moss, James Kerr, Wytske M. van Weerden, Elza C. de Bruin, Shanade Dunn, Brandon Willis, Sarah J. Ross, Claire Rooney, Simon T. Barry

**Affiliations:** 1 Bioscience, Early Oncology, AstraZeneca, Alderley Park, UK; 2 Bioscience, Early Oncology, AstraZeneca, Cambridge, UK; 3 Department of Experimental Urology, Josephine Nefkens Institute, Erasmus University Medical Center, Rotterdam, the Netherlands; 4 Translational Medicine, AstraZeneca, Cambridge, UK; 5 Bioscience, Early Oncology, AstraZeneca, Boston, MA USA

**Keywords:** Drug development, Prostate cancer

## Abstract

**Background/objective:**

To explore the anti-tumour activity of combining AKT inhibition and docetaxel in PTEN protein null and WT prostate tumours.

**Methods:**

Mechanisms associated with docetaxel capivasertib treatment activity in prostate cancer were examined using a panel of in vivo tumour models and cell lines.

**Results:**

Combining docetaxel and capivasertib had increased activity in PTEN null and WT prostate tumour models in vivo. In vitro short-term docetaxel treatment caused cell cycle arrest in the majority of cells. However, a sub-population of docetaxel-persister cells did not undergo G2/M arrest but upregulated phosphorylation of PI3K/AKT pathway effectors GSK3β, p70S6K, 4E-BP1, but to a lesser extent AKT. In vivo acute docetaxel treatment induced p70S6K and 4E-BP1 phosphorylation. Treating PTEN null and WT docetaxel-persister cells with capivasertib reduced PI3K/AKT pathway activation and cell cycle progression. In vitro and in vivo it reduced proliferation and increased apoptosis or DNA damage though effects were more marked in PTEN null cells. Docetaxel-persister cells were partly reliant on GSK3β as a GSK3β inhibitor AZD2858 reversed capivasertib-induced apoptosis and DNA damage.

**Conclusion:**

Capivasertib can enhance anti-tumour effects of docetaxel by targeting residual docetaxel-persister cells, independent of PTEN status, to induce apoptosis and DNA damage in part through GSK3β.

## Introduction

Capivasertib is a selective AKT inhibitor [1] being tested in a number of phase III clinical trials in prostate and breast cancer. In tumour cells, AKT regulates cell proliferation, survival, migration, gene expression and metabolism [2] and is commonly activated via signalling through PI3Kα or β [3]. The PI3K/AKT/mTOR pathway is frequently dysregulated in cancers with activating mutations commonly occuring in *PIK3CA* [4, 5] or *AKT1* [6] activating AKT signalling and rendering cells sensitive to AKT pathway inhibitors [7–10]. Loss of the tumour suppressor PTEN also activates AKT signalling, through PI3Kβ [11–14].

PTEN loss is common in mCRPC [15, 16] and is associated with poorer survival following chemotherapy and hormonal therapy [17, 18]. Monotherapy treatment with PI3K-AKT pathway inhibitors have minimal activity in clinical trials, but improved effects are seen in combination. In prostate cancer, targeting PI3K/AKT signalling by combining PI3Kβ or AKT inhibitors with inhibitors of androgen signalling increases anti-tumour effects in PTEN null tumour cell lines and tumour models due to reciprocal crosstalk between these two pathways [14, 19, 20]. Moreover, combining the AKT inhibitor ipatasertib and abiraterone gave benefit in castrate-resistant prostate cancer with PTEN protein loss [21].

In the Phase II ProCAID clinical study, combining capivasertib and docetaxel improved survival for mCRPC patients regardless of PTEN status [22–24] providing clinical proof of concept that PI3K-AKT inhibition enhances taxane chemotherapy [1, 25–29]. While preclinical work established potential for this combination [30, 31], what drives this broad combination benefit in prostate cancer is poorly understood. Longer treatment of *PIK3CA*, *PTEN* altered gastric cancer models suggests that paclitaxel treated tumour had small increased activation of AKT [25], however, the mechanistic interactions, and shorter time points were not explored. To gain more insight into this combination in prostate cancer we have examined the effects of docetaxel alone and the combination across a panel of xenograft models and cell lines representing PTEN protein null and PTEN protein proficient (PTEN WT) prostate tumours.

## Results

### Capivasertib combines with docetaxel to enhance anti-tumour activity of preclinical prostate cancer models independent of PTEN status

**Fig. 1:**
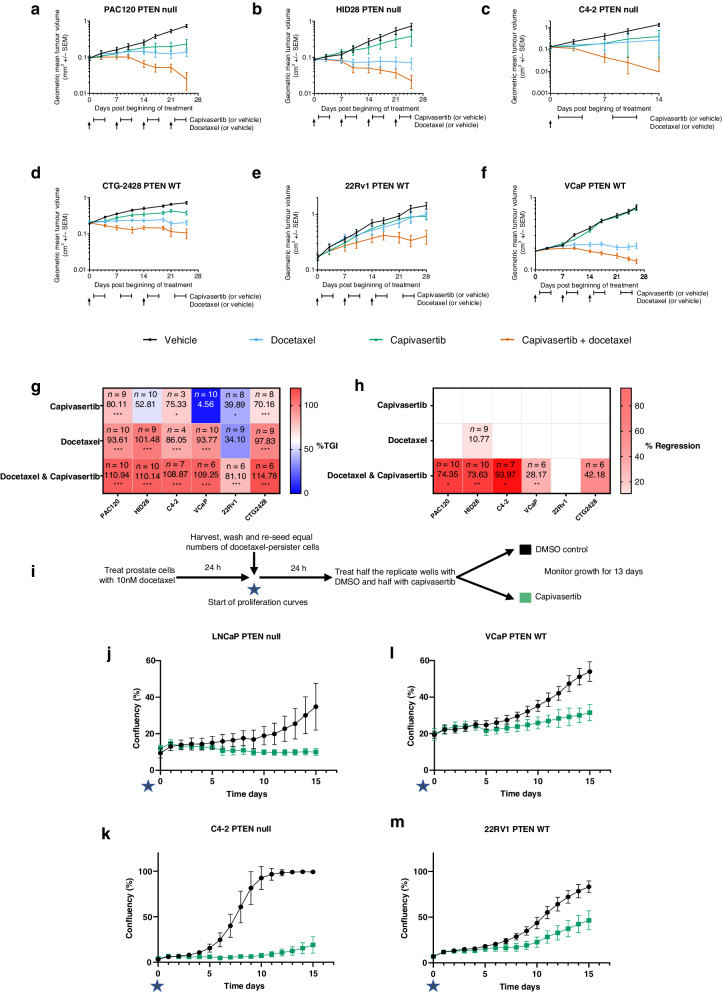
Combining capivasertib with docetaxel shows benefit in PTEN null and PTEN WT prostate cancer tumour and cell line models. Plots showing change in tumour volume over 28 days in PTEN null (**a**, **b**, **c**) and PTEN WT (**d**, **e**, **f**) prostate cancer xenograft models. Docetaxel and capivasertib dosing schedules for each model are indicated (docetaxel dose with upward arrow, capivasertib dose period with square parentheses). Docetaxel cycles were adjusted guided by tolerance studies. PAC120 and HID28 studies were dosed weeks 1 to 4; VCaP and 22Rv1 were dosed weeks 1, 2, and 3; CTG-2428 and C4-2 studies were dosed week 1 and 3. **g** Heatmap depicts % tumour growth inhibition (TGI) scored low (blue) to high (red), numbers show absolute %TGI, with P value versus vehicle control depicted below, *P > 0.05, **P > 0.01, ***P > 0.001. **h** Heatmap depicts % tumour regression scored no regression (white) to high regression (red), numbers show absolute % regression, with *P* value versus vehicle control depicted below, *P > 0.05, **P > 0.01, ***P > 0.001. **i** Schematic of in vitro assay procedure. **j**–**m** Plots showing proliferation of docetaxel or DMSO control pre-treated cells cultured for 13 days in the presence of DMSO control or capivasertib 0.5 µM (LNCaP), 1 µM (C4-2, VCaP and 22RV1). Proliferation is shown as average % confluency from at least triplicate wells. Error bars represent standard deviation. Data are representative of at least two independent experiments.

We have previously shown that capivasertib combines with docetaxel in breast cancer xenografts to enhance anti-tumour activity [1]. To assess the combination of capivasertib and docetaxel more broadly in prostate cancer, a panel of PTEN null and PTEN protein proficient (PTEN WT) prostate PDX and cell line models was used. PDX and xenograft bearing mice were randomised to receive docetaxel (5 mg/kg), capivasertib (130 or 100 mg/kg BID), a combination of docetaxel and capivasertib or treated with the equivalent vehicle controls for up to 28 days. Capivasertib was administered the day after docetaxel injection on 4 days on 3 days off intermittent schedule, mimicking the clinical dosing regimen. Docetaxel cycles for individual models were adjusted guided by tolerance studies (Fig. 1a–f). The combination had broad activity, with increased tumour regression compared to either of the monotherapies, seen in PTEN null (PAC120, HID28, and C4-2), and PTEN WT (CTG-2428 and VCAP) models (Fig. 1a, b, c, d, f, g, h, Supplementary Fig. 1). In an additional PTEN WT (22RV1) model, tumour stasis was achieved (Fig. 1e, g, h, Supplementary Fig. 1). In all models, the combination gave the greatest anti-tumour activity.

The combination of docetaxel and capivasertib was also explored in a proliferation assay in vitro. Prostate cancer cells pre-treated with 10 nM docetaxel for 24 h, were washed and replated prior to incubation with 0.5–1 µM capivasertib or DMSO control for a further 13 days (Fig. 1i). Consistent with the in vivo data, combination benefit was observed with capivasertib treatment delaying outgrowth of docetaxel pre-treated cells (Fig. 1j–m).

In summary, these data show that in preclinical PTEN null and PTEN WT prostate cancer xenograft tumour models and cell lines, addition of capivasertib following docetaxel pre-treatment improved the anti-tumour effects.

### Short term docetaxel treatment is associated with increased phosphorylation of PI3K/AKT pathway effectors in preclinical prostate cancer models

To study the mechanism driving the combination benefit in vitro in more detail, we first investigated the effect of short-term treatment with docetaxel monotherapy on the cell cycle. PTEN null (PC3, LNCaP, PC346Flu1) and PTEN WT (22RV1, VCaP) prostate cancer cells were treated for 24 h with DMSO control, 10 and 100 nM docetaxel, to mimic the ranges of exposure observed clinically [32, 33]. Following treatment of cultured cells with docetaxel two distinct populations of cells were present, suspension cells and adherent cells. Due to the phenotypic differences between these two populations, the suspension and adherent populations were analysed separately. Flow cytometry cell cycle status of suspension populations indicated increased numbers of cells in subG1 and high phospho-histone H3 positive G2 cells, indicative of cell death and G2/M arrest respectively and consistent with docetaxel sensitivity [34]. In contrast, fewer adherent cells were in subG1 and G2/M arrest and more in G1 and active S phase (Supplementary Table 1, Supplementary Results). Suspension and adherent cell fractions were also analysed by immunoblotting (Supplementary Fig. 2A, B). Quantification of the ratio of cleaved:full length PARP indicated that in VCaP cells greater PARP cleavage associated within the suspension cell populations following 24 h treatment with 10 and 100 nM docetaxel whilst in LNCaP cells greater PARP cleavage associated with the suspension cell population following treatment with 100 nM docetaxel (Supplementary Fig. 2A) indicating increased apoptosis in these cells. Similarly, the trend of higher levels of apoptosis in suspension cells was observed in PTEN null (PC3 and C4-2) and PTEN WT (22RV1) cells treated with 10 and 100 nM docetaxel (Supplementary Fig. 2B). Taken together the lower level of apoptosis and G2/M arrest observed in the adherent cells suggested they survived 24 h docetaxel treatment and represent a population of short-term docetaxel-persister cells. We therefore focused our studies on docetaxel-persister cells to explore the combination in vitro.

Previous studies suggest induction of phosphorylated AKT in gastric and prostate cells treated with docetaxel [25, 30, 35], here we investigate the effects of short-term docetaxel treatment on phosphorylation of various substrates downstream of AKT in prostate cells.

**Fig. 2:**
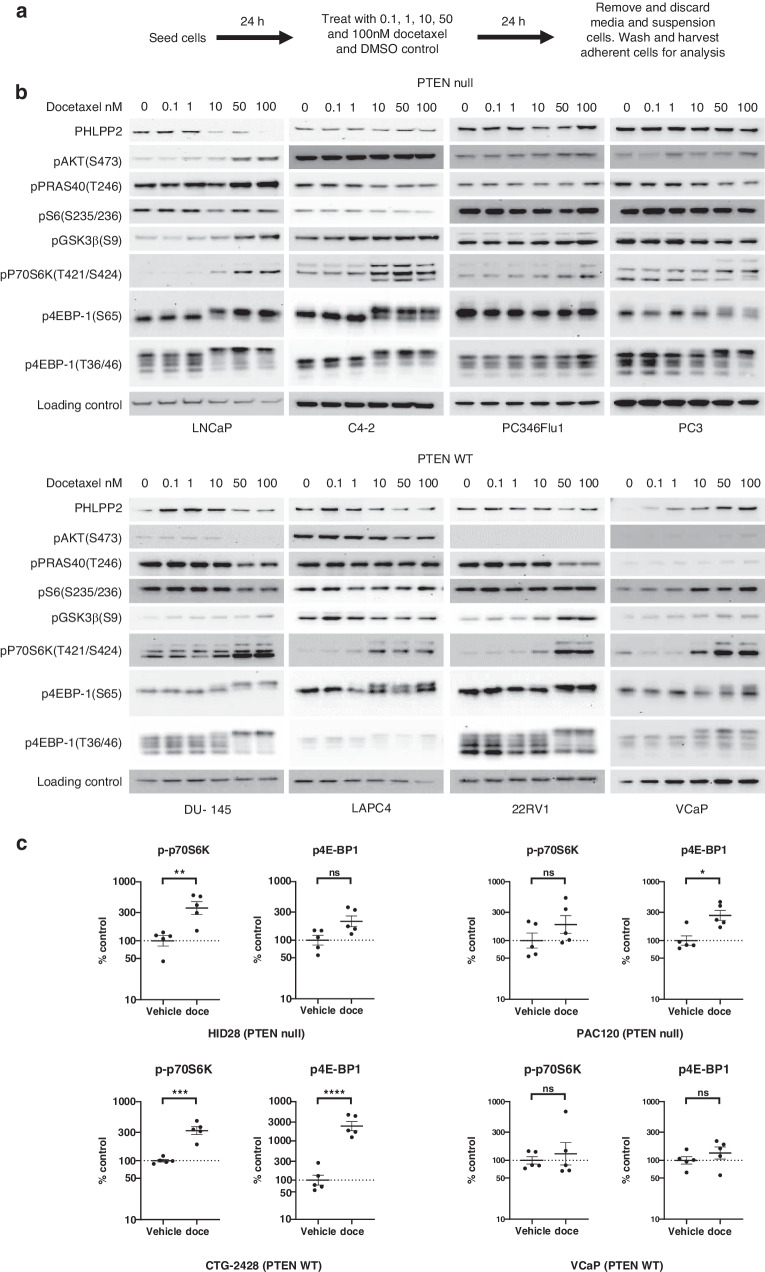
Short term docetaxel treatment is associated with increased phosphorylation of PI3K/AKT pathway effectors in preclinical prostate cancer models. **a** Schematic of the assay procedure. **b** Western blot profile of indicated PI3K/AKT pathway markers in lysates from adherent docetaxel-persister PTEN null and PTEN WT prostate cancer cells treated for 24 h with 0.1, 1, 10, 50, and 100 nM docetaxel or DMSO control. Loading control: Vinculin (LNCaP, C4-2); GAPDH (PC346 Flu1, PC3, DU145, LAPC4, 22RV1, VCaP). **c** Pharmacodynamic changes in phosphorylation levels of p70S6K (T421/S424) and hyperphosphorylated levels of 4EBP1 in PTEN null (HID28 and PAC120) and PTEN WT (CTG-2428 and VCaP) xenograft models. Tumours were collected from animals on day 4 after one dose of docetaxel on day 1. Data normalised to the geomean of β-actin and shown as percentage change from control plotted as mean ± SEM (*n* = 5). Statistical analysis ANOVA test vs vehicle treated, *p < 0.05; **p < 0.01; ***p < 0.005, ****p < 0.001.

A panel of PTEN null and PTEN WT prostate cancer cell lines were treated with increasing concentrations of docetaxel for 24 h and PI3K/AKT pathway activation analysed in the docetaxel-persister cells by immunoblotting (Fig. 2a, b). Across the panel of cell lines tested, differential responses to docetaxel treatment were observed at the level of p-AKT(S473). In PTEN null cells induction of p-AKT(S473) was only observed in LNCaP cells, while in DU145 and LAPC4 PTEN WT cells p-AKT(S473) was reduced following docetaxel treatment and no p-AKT(S473) was detected in 22RV1 and VCAP cells. However, modulation of the PI3K/AKT pathway downstream of AKT including phosphorylation of GSK3β, p70S6K and 4E-BP1 was observed. Docetaxel treatment induced p-GSK3β(S9) in a dose dependent manner in PTEN null (LNCaP) and PTEN WT (DU145, 22RV1 and VCaP) cell lines and at 100 nM docetaxel only in PC346Flu1 cells. Cell lines in which docetaxel treatment did not result in a dose dependent induction of pGSK3β(S9) (C4-2, PC3, LAPC4 and PC346Flu1) had higher basal p-GSK3β(S9) levels. In all cell lines docetaxel treatment induced p-p70S6K(T421/S424) levels and hyperphosphorylation of p-4E-BP1(T37/46). The identity of these hyperphosphorylated 4E-BP1 species was not investigated in detail, however phosphorylation of 4E-BP1 at additional sites is associated with mitosis, altered protein translation and polyploidy [36–38]. Docetaxel induction of p-p70S6K(T421/S424) and p-4E-BP1(T37/46) varied between cell lines with 10 nM sufficient in LNCaP, C4-2, DU145, LAPC4 and VCaP cells and 50 to 100 nM required in PC346-Flu, PC3 and 22RV1 cells. The induction of p-p70S6K(T421/S424) and 4E-BP1 hyperphosphorylation following a single dose of docetaxel in vivo was also investigated (Fig. 2c). Docetaxel treatment induced a significant increase in p-p70S6K(T421/S424) levels in PTEN null (HID28) and PTEN WT (CTG-2428) tumours and a trend to higher levels in PTEN null (PAC120) tumours. Similarly, docetaxel treatment resulted in hyperphosphorylation of 4E-BP1 in PTEN null (HID28 and PAC120) tumours and PTEN WT (CTG-2428) tumours. Changes in phosphorylation on p70S6K and 4E-BP1 were not observed in VCaP tumours.

### Capivasertib reverses PI3K/AKT pathway activation and alters cell cycle progression in short-term-docetaxel-persister cells

**Fig. 3:**
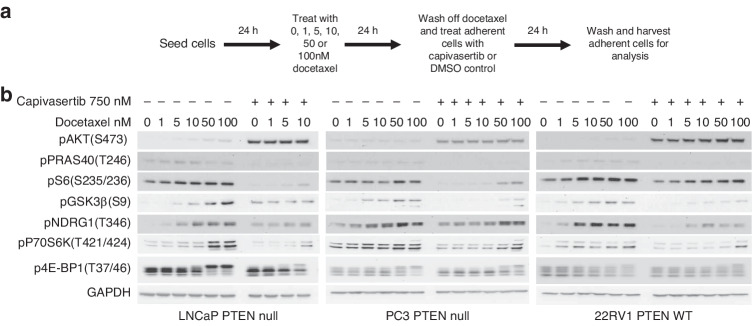
Capivasertib reverses PI3K/AKT pathway activation and alters cell cycle progression in short-term-docetaxel-persister cells. **a** Schematic of the assay procedure. **b** Western blot analysis of indicated PI3K/AKT pathway markers in lysates from adherent PTEN null and PTEN WT cells pre-treated with dose titrations of docetaxel for 24 h followed by wash off and subsequent treatment with 750 nM capivasertib for 24 h. Data are representative of two replicate experiments.

Having established that 24 h docetaxel exposure can induce phosphorylation of the AKT downstream pathway markers GSK3β, p70S6K and 4E-BP1 in short-term-docetaxel-persister cells, the effect of capivasertib was examined. LNCaP, PC3 and 22RV1 cells were treated with increasing concentrations of docetaxel for 24 h. Suspension cells were removed and remaining adhered docetaxel-persister cells were washed with PBS prior to treatment with capivasertib or DMSO control for a further 24 h (Fig. 3a, b). In DMSO control cells the dose dependent induction of phosphorylation of GSK3β and p70S6K by docetaxel was maintained in LNCaP, PC3 and 22RV1 cells 24 h after the removal of docetaxel suggesting prolonged docetaxel effects (Figs. 2b and 3b). Addition of capivasertib (750 nM) to PTEN null (LNCaP and PC3) and PTEN WT (22RV1) cells decreased levels of phosphorylated p70S6K1, GSK3β, S6, NDRG-1 and PRAS40 confirming that phosphorylation of these proteins in docetaxel-persister cells is AKT dependent (Fig. 3b).

Next, the impact of the monotherapy and combination treatments on cell cycle in the docetaxel-persister cells was analysed by flow cytometry using the same treatment schedule (Supplementary Fig. 3A, B). Capivasertib monotherapy treatment increased the percent of cells in sub-G1/G1 in cells sensitive to monotherapy treatment consistent with previous reports [19]. The combination treatment reduced the total number of adherent cells relative to docetaxel monotherapy treatment (Supplementary Fig. 3A) however, the percentage of cells in each cell cycle phase was similar (Supplementary Fig. 3B). Western blot analysis for expression of proteins associated with cell cycle control showed that in PTEN null (C4-2, LNCaP, and PC3) and PTEN WT (22RV1 and VCaP) cells combination treatment reduced levels of cyclin B1 (indicative of mitotic exit) and reduced cyclin D1 and/or phospho-Rb (S807/811) levels (indicative of G1 arrest) (Supplementary Fig. 4A). Docetaxel induced total p21 levels in p53 functional, (C4-2, LNCaP, PC345Flu1 and 22RV1) and p53 non-functional (PC3 and VCaP) cells whereas capivasertib treatment alone or in combination, decreased p21 levels and induced p27 in C4-2, LNCaP and PC346Flu1 cells, consistent with p27 being a direct target of AKT [39, 40], (Supplementary Fig. 4A). Reduced p21 and increased p27 expression has previously been associated with a senescence-like phenotype following PI3K/AKT pathway inhibition [41]. However, induction of the senescence marker beta-galactosidase [42] was not observed in C4-2, LNCaP or PC346Flu1 docetaxel-persister cells treated with capivasertib or the combination (Supplementary Fig. 4B).

Taken together, the data indicate that capivasertib treatment of prostate docetaxel-persister cells reduces G2 arrest and inhibits further cycling.

### Capivasertib enhances apoptosis in docetaxel-persister cells in a GSK3β dependent manner

**Fig. 4:**
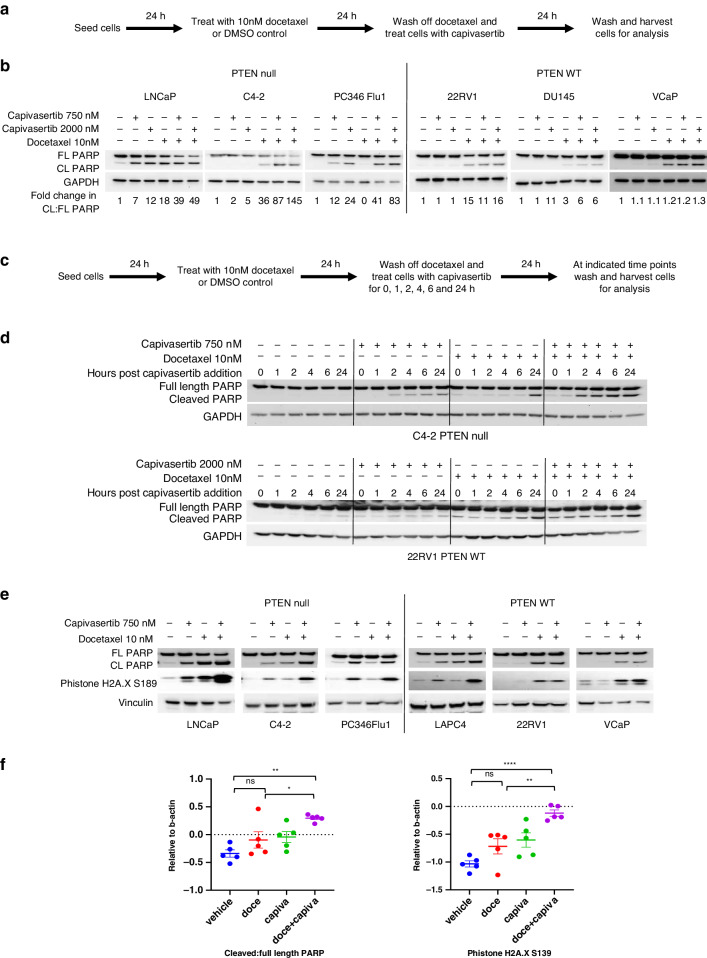
Capivasertib enhances apoptosis in docetaxel-persister cells. **a** Schematic of the assay procedure. **b** Western blot analysis of full length and cleaved PARP in lysates from adherent PTEN null and PTEN WT cells pre-treated with 10 nM docetaxel or DMSO control for 24 h followed by wash off and subsequent treatment with 750 and 2000 nM capivasertib or DMSO control for 24 h. Cleaved and full-length bands were quantified using GeneTools (Syngene) and are shown for each sample as fold change relative to DMSO control. Data are representative of two similar experiments. **c** Schematic of the assay procedure for apoptosis induction over time. **d** Western blot analysis of full length and cleaved PARP in lysates from adherent PTEN null and PTEN WT cells pre-treated with 10 nM docetaxel or DMSO control were washed and treated with 750 nM (C4-2) or 2000 nM (22RV1) capivasertib for indicated time intervals over 24 h. Data are representative of two identical experiments. **e** Western blot analysis of markers of apoptosis and DNA damage in lysates from adherent PTEN null and PTEN WT cells pre-treated with 10 nM docetaxel or DMSO control for 24 h followed by wash off and subsequent treatment with 750 and 2000 nM capivasertib or DMSO control for 24 h. **f** Pharmacodynamic changes in levels of cleaved:full length PARP and p-histone H2A.X in the PTEN null (HID28) xenograft model. Tumours were collected from animals on day 4 after one dose of docetaxel or vehicle control on day 1 followed by capivasertib or vehicle control dosed BID on days 2–4. Data are normalised to the geomean of β-actin and shown as percentage change from control plotted as mean ± SEM (*n* = 5). Statistical analysis ANOVA test vs vehicle treated, *p < 0.05; **p < 0.01; ***p < 0.005, ****p < 0.001.

As the combination treatment consistently reduced the number of docetaxel-persister cells relative to docetaxel pre-treatment alone (Supplementary Fig. 3A) and did not induce senescence (Supplementary Fig. 4B) induction of apoptosis was assessed as a potential mode of action. The induction of apoptosis was assessed by western analysis of full length and cleaved PARP. Across the panel of PTEN null and PTEN WT prostate cell lines, treatment of docetaxel-persister cells with 750 and 2000 nM capivasertib, increased the ratio of cleaved to full length PARP compared to docetaxel monotherapy in C4-2, LNCaP, PC346Flu1 and DU145 cells and with 2000 nM capivasertib in 22RV1 and VCaP cells (Fig. 4b) consistent with an induction of apoptosis in the docetaxel-persister cells.

Kinetics of apoptosis induction were further explored in vitro in docetaxel-persister cells pre-treated for 24 h with 10 nM docetaxel (Fig. 4c). In C4-2 cells the steady induction of cleaved PARP over 24 h was rapidly increased on the addition of capivasertib with robust induction at 2 h post-dose and to higher levels than that observed with capivasertib monotherapy. Similar trends were seen in the 22RV1 docetaxel-persister cells, however higher levels of cleaved PARP were observed in this model already with docetaxel monotherapy (Fig. 4d). Consistent with the induction of apoptosis, docetaxel mediated induction of p21, which has previously been associated with resistance to apoptosis [43], was reversed by capivasertib treatment in C4-2, LNCaP, PC346Flu1 and VCaP cells (Supplementary Fig. 4A). In addition, the combination inreased levels of p-histone H2A.X(S189), indicative of DNA damage, in PTEN null (LNCaP, C4-2 and PC346Flu1) and PTEN WT (LAPC4 and VCaP) cells above levels observed with either monotherapy (Fig. 4e). Consistent with the in vitro observations a significant increase in levels of cleaved PARP and p-histone H2A.X(S189) were observed in vivo in PTEN null (HID28) tumours treated with a single dose of docetaxel on day 1 and with capivasertib on days 2–4 (Fig. 4f). Taken together these data suggest that capivasertib treatment increases the level and rate of apoptosis of docetaxel-persister prostate cancer cells.

**Fig. 5:**
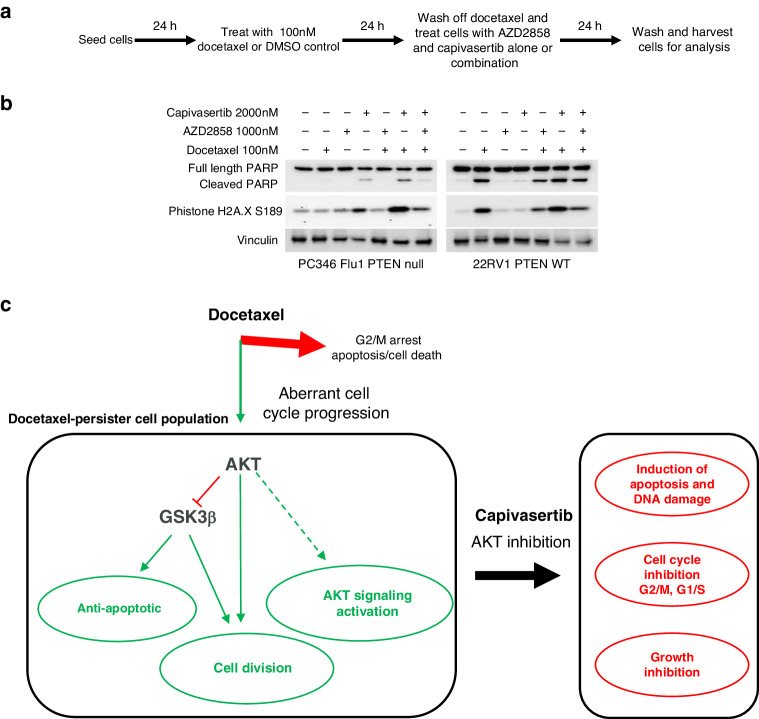
Capivasertib enhances apoptosis in docetaxel pre-treated cells in a GSK3 dependent manner. **a** Schematic of the assay procedure. **b** Western blot analysis of indicated markers in lysates from PTEN null and PTEN WT cells pre-treated 100 nM docetaxel for 24 h followed by wash off and subsequent treatment with 1000 nM AZD2858 and 2000 nM capivasertib alone and in combination for 24 h. Data shown are representative of two similar experiments. **c** Schematic summarising the effects of capivasertib on the growth and survival of docetaxel-persister cells.

The association of AKT pathway inhibition with increased apoptosis was further investigated. Phosphorylation of GSK3β (associated with inhibition of GSK3β activity) promotes cell survival and evasion of apoptosis [44, 45]. Phospho-GSK3β(S9) was increased by 24 h docetaxel treatment in a dose dependent manner in LNCaP, C4-2, DU145, 22RV1 and VCaP cells (Fig. 2b). Therefore, the possibility that reducing GSK3β activity following docetaxel treatment may contribute to survival by mediating resistance to docetaxel induced cell death was tested. Reduction of pGSK3β(S9) in LNCaP and PC346Flu1 docetaxel-persister cells treated with capivasertib associated with increased cleaved PARP (Supplementary Fig. 5A, B). To prevent GSK3β mediated survival a GSK3α/β inhibitor (AZD2858) was used. AZD2858 treatment resulted in maximum inhibition of the GSK3β substrate pTau(T205) at 1 µM in 22RV1 cells (Supplementary Fig. 6A, B). PTEN null (C4-2 and PC346Flu1) and PTEN WT (22RV1) cells pre-treated with docetaxel were subsequently treated with AZD2858 and capivasertib as monotherapy or in combination (Fig. 5a, Supplementary Fig. 6A). To varying degrees, capivasertib monotherapy induced apoptosis in all three cell lines (Fig. 5a, Supplementary Fig. 6C). As previously observed, addition of capivasertib enhanced apoptosis in docetaxel-persister cells above that seen with capivasertib or docetaxel treatment alone. Increased induction of apoptosis by capivasertib was observed in C4-2 docetaxel-persister cells pre-treated with <10 nM of docetaxel and in PC346Flu1 and 22RV1 docetaxel-persister cells pre-treated with higher docetaxel concentrations (Fig. 5a, Supplementary Fig. 6C). AZD2858 monotherapy treatment had no effect on apoptosis in these cells. However, when docetaxel-persister cells were treated with capivasertib and AZD2585 in combination, the induction of apoptosis observed with capivasertib was reduced (Fig. 5b, Supplementary Fig. 6C). In addition, the induction of p-histone H2A.X(S189) and the reduction in cyclin D1 and p21 levels observed with capivasertib monotherapy in docetaxel-persister cells were reversed with the combination of AZD2858 and capivasertib suggesting that GSK3β activity may contribute to the cell cycle inhibitory effects of capivasertib on docetaxel-persister cells (Fig. 5b, Supplementary Fig. 6C). Taken together the data suggests that capivasertib inhibits proliferation and survival of persister cells remaining after docetaxel treatment via modulation of AKT downstream substrates including GSK3β (Fig. 5c).

## Discussion

Here, we show that combining the AKT inhibitor capivasertib with docetaxel increases anti-tumour effects in PTEN null and PTEN WT prostate tumour models and cell lines. In vivo the combination increased tumour growth inhibition in all models assessed, with significant tumour regressions in 3/6 models. In vitro sequential addition of capivasertib following short-term (24 h) docetaxel incubation reduced cell growth in PTEN null and WT cells. In vitro, acute docetaxel treatment killed a substantial number of cells and induced G2/M arrest. However, in a residual docetaxel-persister cell fraction that remained adherent to the plastic, a dose-dependent increase in phosphorylation of p70S6K, 4E-BP1 and in some cell lines, GSK3β occurred, which was reversed by capivasertib. These cells had progressed through G2/M arrest and remained in cycle without cell division. Generally, capivasertib monotherapy induces a G1/S arrest, and in combination capivasertib reduced cell cycle in docetaxel-persister cells. Moreover in 5/7 cell lines combination treatment increased apoptosis and induction of DNA damage. The fact that the increased apoptosis induced by capivasertib was reversed or reduced by inhibiting GSK3β suggests a direct role for AKT signalling. Although the PI3K/AKT inhibitor taxane combination is effective in preclinical models [1, 46, 47], PI3K-AKT pathway inhibition prior to taxane reduces efficacy as the PI3K-AKT mediated G1/S arrest blocks progression through S phase and G2 where taxanes induce cell death [26]. Inhibiting AKT post-taxane treatment increased tumour growth inhibition in breast, gastric and prostate cancer models [1, 25, 27, 29].

It has been suggested that long term resistance to docetaxel is associated with an increase in AKT phosphorylation [25, 30, 35]. Here in PTEN WT and PTEN null prostate cancer cells, following acute treatment with docetaxel the increased AKT phosphorylation in the docetaxel-persister cells was less apparent in the cell lines tested. Commonly phosphorylation of p70S6K(T421/S424) and 4E-BP1(T37/46), downstream of AKT, was observed. Induction of phospho-GSK3β (S9) was also observed with docetaxel treatment in some cell lines, while in cell lines where increased phosphorylation of GSK3β was less apparent baseline phospho-GSK3β levels were high prior to treatment. Interestingly increased phosphorylation of S6 was not seen in all cell lines, which may indicate that increased PI3K-AKT pathway activation in docetaxel-persister cells influences cell cycle or survival rather than general PI3K-mTOR activation including effects on protein synthesis [36, 48]. Modulation of 4E-BP1 also suggests a cell cycle or cell stress response following docetaxel treatment [37, 38]. One other study has examined the acute response to docetaxel in ER+ breast cancer MCF7 cells where a transient increase in pAKT was observed [49]. However, the induction of signalling downstream of AKT in the absence of increased pAKT signalling has been observed in other settings. In ER+ breast cancer cell lines that have become oestrogen independent following long term oestrogen deprivation, or resistant to the CDK4/6 inhibitor palbociclib increases in pS6 and other markers downstream of AKT are observed but little pAKT is detected or minimal to no change in pAKT [50, 51]. The significance of this warrants further investigation. While the data show persister cells are impacted by capivasertib treatment, we have not performed unbiased phospho-site profiling or reverse phase protein array phospho analysis to look at all changes following docetaxel and combination treatment, therefore changes in other proteins or pathways may be associated with survival of the docetaxel persister cells.

It was clear that across a panel of tumour cells the response to docetaxel and hence the combination was heterogeneous. In vitro combination activity was enhanced in cells with monotherapy sensitivity to capivasertib. However, in vivo additive anti-tumour combination effects were seen in 5 out of 6 models and appeared related to the intrinsic response of tumour models to docetaxel. In models that were more sensitive to docetaxel, capivasertib addition drove regressions, whereas in less sensitive models the combination resulted in cytostatic effects.

Inhibition of AKT signalling can contribute to combination benefit through different mechanisms, and it is possible that more than one mechanism is important in a specific cell line or tumour model. AKT-mediated phosphorylation of GSK3β, p70S6K and 4E-BP1, can enable evasion of apoptosis, cell cycle progression in the face of a G2/M blocker and absence of cell division. These mechanisms may not always be induced by docetaxel, cells may have higher baseline signalling, contributing to both intrinsic and induced resistance that is reduced by capivasertib treatment. For example, high intrinsic GSK3β activity could render docetaxel less effective independent of other PI3K pathway functions. While GSK3β may be an important mediator of persistence following docetaxel treatment, other mechanisms may also contribute, e.g. changes in translation downstream of mTORC1, coupling through 4E-BP1 or alternate non-canonical p70S6K signalling. For example, it is possible that intrinsic activity of AKT in the context of the cell cycle status of the persister cell fraction drives the survival effect. Alternatively pAKT increases may be more transient than the increase seen in GSK3β and p70S6K, but phosphorylation is still regulated by AKT or finally that docetaxel induced cell stress dysregulates phosphatases such as PTEN and PHLPP that control AKT [52, 53]. In addition TSC1, TSC2 [54], and PP2A [55] that regulate P70S6K could also be disrupted which may reduce the activation threshold for signalling molecules down stream of AKT. Indeed, in breast cancer cells reduction of mTORC1-4EBP1 signalling results in a reduction in PTEN protein [56]. It will be important to further evaluate whether other mechanisms that are regulated directly or indirectly by AKT modulation by capivasertib also make a contribution to the combination benefit. Variable sensitivity of prostate cancer cells to taxanes can also be influenced by differences in drug uptake and consequent intracellular levels of drug [57]. Therefore, different features may be important in different cells, and it is likely there is not one single unifying mechanism driving combination benefit. For example, it is possible that on long term treatment there may be engagement of the immune system, changes in the tumour microenvironment (TME) or adaptive responses in the TME, or the tumour cells.

Here, we have examined the acute interaction between docetaxel and capivasertib and sought to mimic the Phase II ProCAID study schedule [23, 24] in vivo and in vitro, but have not assessed prevention of longer-term resistance. The data presented here support the ProCAID study observation that the combination could be broadly effective in prostate cancer. How the pathway is activated remains an interesting question. It may be as a result of cells being in a specific phase of the cell cycle when treated with docetaxel, through inactivation of phosphatase regulation following redox stress, or signalling through other pathways such as activation of DNA damage repair proteins in response to aberrant mitosis [58].

In summary, combining the AKT inhibitor capivasertib with docetaxel in prostate cancer improves anti-tumour effects by targeting the residual surviving cells following docetaxel treatment. The benefit can be driven through different mechanisms downstream of AKT, by reducing AKT mediated cell cycle progression and enhancing induction of apoptosis or DNA damage in cells that persist after docetaxel treatment.

## Methods and materials

### Cell lines, cell culture and compound reagents

All AstraZeneca cell lines were authenticated by short-tandem repeat analysis (STR). VCaP (CRL-2876), 22RV1 (CRL-2505), LNCaP (CRL-1740) and PC3 (CRL-1435) cells were obtained from ATCC. DU145 (ACC-298) cells were obtained from DSMZ. C4-2 cells were from Professor George N. Thalmann, University of Bern; LAPC4 cells were from Dr. Beth, Indiana University and PC346 Flu1 cells were from Dr. van Weerden, Erasmus University Medical Center, Netherlands. C4-2, LAPC4, LNCaP, PC3, VCaP and 22RV1 cells were cultured in RPMI (phenol red free) (ThermoFisher Scientific) containing 10% FBS (ThermoFisher Scientific) with 2 mmol/L GlutaMAX Supplement (ThermoFisher Scientific). PC346Flu1 cells were cultured in DMEM/F12 media (ThermoFisher Scientific), 2% Hyclone FBS charcoal stripped (Sigma) plus supplements as previously described [59]. All assays were carried out on cell cultures with 70–80% confluency. Capivasertib, docetaxel, hydroxyflutamide and AZD2858 were synthesised according to published methods.

### In vivo studies

Full methods are detailed in Supplementary information. All animal work was conducted according to AstraZeneca’s Global Bioethics Policy (https://www.astrazeneca.com/content/dam/az/Sustainability/Bioethics_Policy.pdf), in accordance with the PREPARE and ARRIVE guidelines. For efficacy studies (*n* = 10 animals per arm, dosing for up to 4 weeks) animals were dosed with; 100 mg/kg (PAC120, HID28, VCaP, CTG2428 and C4-2) or 130 mg/kg (22RV1) BID capivasertib (4 days on 3 days off, starting the day after docetaxel dosing, in 10% DMSO/25% Kleptose, pH5, 10 ml/kg); 5 mg/kg Docetaxel (up to once weekly, starting the day before capivasertib (PAC120 and HID28 dosed weeks 1-4; 22RV1 and VCaP dosed weeks 1-3; CTG-2428 and C4-2 dosed weeks 1 and week 3), in Physiological Saline, 10 ml/kg); a combination of capivasertib and Docetaxel (as above); or treated with equivalent vehicle controls. Dosing schedules for each model are highlighted in Fig. 1. For docetaxel monotherapy pharmacodynamic studies (*n* = 5 animals per arm) animals received (PAC120, HID28, VCaP andCTG2428) 5 mg/kg IV Docetaxel once, starting on day 1. On Day 4, mice were humanely euthanised and tumour tissue collected and immediately snap frozen in liquid nitrogen before storage at −80 °C. For HID28 combination pharmacodynamic studies (*n* = 5 animals per arm) animals were dosed over a 4 day period, with sampling + 4 h relative to the morning dose on day 4. Animals received 100 mg/kg PO capivasertib (2 days BID with an additional AM dose on the 3rd day, starting the day after docetaxel dosing, in 10% DMSO/25% Kleptose, pH5, 10 ml/kg); 5 mg/kg IV Docetaxel (once, starting on day 1, the day before capivasertib. Docetaxel dosed in Physiological Saline, 10 ml/kg); a combination of capivasertib and Docetaxel (as above); or treated with equivalent vehicle controls. On Day 4 mice were humanely euthanised and tumour tissue collected and immediately snap frozen in liquid nitrogen before storage at −80 °C. For all samples snap frozen tumour fragments underwent protein extraction by adding 900 μL of Extraction buffer (20 mM Tris (pH 7.5) #Sigma T2319, 137 mM NaCl #Sigma S5150, 10% Glycerol #Sigma G5516, 50 mM NaF #Sigma S6776, 1 mM Na3VO4 #Sigma S6508, 1% SDS, 1% NP40 substitute Roche #11754599001) with complete protease inhibitor cocktail (Roche #11836145001; 1 tablet per 50 mL) and phosphatase inhibitor cocktail #3 (Sigma #P0044) with benzonase nuclease (Sigma E1014). Samples were homogenised for 30 s three times at 6.5 m/s in fast-prep machine with an incubation at 4 °C for 5 min between runs. Lysates were then sonicated in chilled diagenode bioruptor in chilled water bath for five cycles of 30 s on high/30 s off. Lysates were then centrifuged for 10 min at 13,000 rpm at 4 °C for two times, with a change of tube between runs to discard debris. Lysates were transferred into a new tube, and protein in the supernatant measured (Thermofisher #23227). Lysates were analysed by western blot as outlined in the immunoblotting methods.

### In vitro 13-day proliferation assay using short-term-docetaxel-persister cells

LNCaP, C4-2, 22RV1 and VCaP prostate cells were treated with 10 nM docetaxel for 24 h. Suspension cells were removed and remaining docetaxel-persister cells washed twice with PBS, trypsinised and harvested and the number of viable cells determined using Trypan Blue reagent. The docetaxel-persister cells were replated in a 384 well plate at 2000 live cells per well for LNCaP, C4-2 and 22RV1 and 5000 live cells per well for VCaP cells across at least eight replicate wells. The plate was placed in an Incucyte S3 Live-Cell Analysis Instrument (Sartorius) and imaged every 4 h. The following day the cells were dosed with DMSO control or capivasertib across four replicate wells per treatment. Imaging was monitored for 13 days. Every 3–4 days half of the media from each well was removed and fresh treatment added. Data was measured as confluency and plotted using the Incucyte software.

### Immunoblotting

Treated cells were washed with PBS and protein extracted at room temperature with RIPA lysis buffer supplemented with HALT protease and phosphatase inhibitors and EDTA (ThermoFisher Scientific). Total cellular protein was separated on precast 4–12% Bis-Tris polyacrylamide gels (Invitrogen) and transferred to membranes. Membranes were blocked in 5% (w/v) non-fat milk in Tris-buffered saline containing 0.05% Tween-20, and then probed with primary antibodies diluted in the same buffer overnight at 4 °C. After washing and incubation with secondary antibodies, blots were incubated with horseradish peroxidase Western Lightning substrate (Perkin Elmer) or SuperSignal West Femto Maximum Sensitivity Substrate (Thermofisher Scientific) according to the manufacturer’s instructions, followed by visualisation of immunoreactivity. Antibody and dilution details are shown in Supplementary Table 2.

### Supplementary information

Supplementary Information

Supplementary Figures
