# Supplementary material for: Capivasertib combines with docetaxel to enhance anti-tumour activity through inhibition of AKT-mediated survival mechanisms in prostate cancer
Source: Br J Cancer. 2024 Feb 23;130(8):1377–87. doi: 10.1038/s41416-024-02614-w (PMC11014923; doi:10.1038/s41416-024-02614-w)
Supplement: Supplementary file 1 — Supplementary Information [file 41416_2024_2614_MOESM1_ESM.docx]

Supplementary information: Capivasertib combines with docetaxel to enhance anti-tumour activity through inhibition of AKT mediated survival mechanisms in prostate cancer

Cath Eberlein^1^, Stuart C Williamson^2^, Lorna Hopcroft^2^, Susana Ros^2^, Jennifer Moss^2^, James Kerr^2^, Wytske M. van Weerden^5^, Elza C de Bruin^3^, Shanade Dunn^2^, Brandon Willis^4^, Sarah Ross^2^, Claire Rooney^3^, Simon T Barry^2^

^1^Bioscience, Early Oncology, AstraZeneca, Alderley Park, UK.

^2^Bioscience, Early Oncology, AstraZeneca, Cambridge, UK.

^3^Translational Medicine, AstraZeneca, Cambridge, UK.

^4^Bioscience, Early Oncology, AstraZeneca, Boston USA.

^5^Department of Experimental Urology, Josephine Nefkens Institute, Erasmus University Medical Center, Rotterdam, Netherlands.

**Supplementary Tables**

**Supplementary Table 1** Summary of the effects of docetaxel on cell cycle status in prostate cancer cell lines.


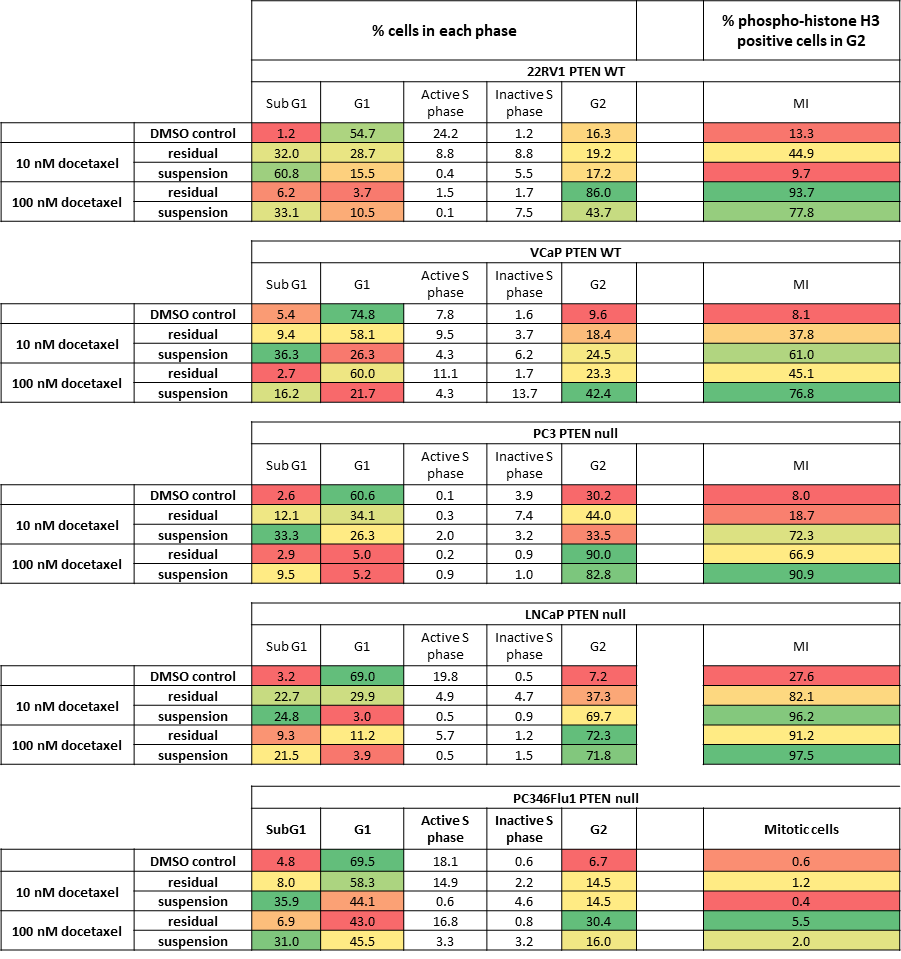


Values represent the percentage for each cell population, in each phase of the cell cycle. Mitotic index (MI) is the percentage of cells within G2 positive for phospho-histone H3 staining. Data shown is representative of two replicate experiments.

**Supplementary Table 2.** Details for antibodies used in immunoblotting experiments.

| **Target** | **Cat. No** | **Supplier** | **dilution** | **Source** | **Size kDa** |
| --- | --- | --- | --- | --- | --- |
| PHLPPL | 25244-1-AP | Proteintech | 1:1000 | Rabbit | 147 |
| Phospho-Akt (Ser473) (D9E) XP | 4060 | CST | 1 in 1000 | Rabbit | 60 |
| Phospho-PRAS40 (Thr246) (D4D2) XP | 13175 | CST | 1 in 1000 | Rabbit | 40 |
| Phospho-S6 Ribosomal Protein (Ser235/236) XP | 4858 | CST | 1 in 1000 | Rabbit | 32 |
| Phospho-GSK-3β (Ser9) (5B3) | 9323 | CST | 1:1000 | Rabbit | 46 |
| pP70S6K(Thr421/Ser424) | 9204 | CST | 1:1000 | Rabbit | 70, 85 |
| p4EBP1(S65) | 9451 | CST | 1:1000 | Rabbit | 16-18 |
| p4EBP1(T37/46) | 9459 | CST | 1:1000 | Rabbit | 16-18 |
| GAPDH (D16H11) XP | 5174 | CST | 1:1000 | Rabbit | 37 |
| Vinculin | V9131 | Sigma | 1:2000 | Mouse | 116 |
| Phospho-NDRG (Thr246) | 5482 | CST | 1:1000 | Rabbit | 46/48 |
| Cyclin A2 (E1D9T) | 91500 | CST | 1:1000 | Rabbit | 55 |
| Cyclin B1 (D5C10) XP | 12231 | CST | 1:1000 | Rabbit | 55 |
| Cyclin D1 | 2922 | CST | 1:1000 | Rabbit | 36 |
| Cyclin E1 (HE12) | 4129 | CST | 1:1000 | Mouse | 48 |
| p21 Waf1/Cip1 (12D1) | 2947 | CST | 1:1000 | Rabbit | 21 |
| Phospho-Rb (Ser807/811) (D20B12) | 8516 | CST | 1:1000 | Rabbit | 110 |
| Rb (4H1) | 9309 | CST | 1:1000 | Mouse | 110 |
| p27 Kip1 (D69C12) XP | 3686 | CST | 1:1000 | Rabbit | 27 |
| PARP | 9542 | CST | 1:1000 | Rabbit | 116, 89 |
| Androgen Receptor (D6F11) XP | 5153 | CST | 1:1000 | Rabbit | 110 |
| P53 (DO-1) | 180325 | CST | 1:1000 | Mouse | 53 |
| Phospho-Histone H2A.X (Ser139) | 2577 | CST | 1:1000 | Rabbit | 15 |

**Supplementary materials and methods**

***In vivo* studies**

All animal work was conducted according to AstraZeneca’s Global Bioethics Policy (<https://www.astrazeneca.com/content/dam/az/Sustainability/Bioethics_Policy.pdf>), in accordance with the PREPARE and reported in line with the ARRIVE guidelines.

PAC120 and HID28 studies were performed under contract with Xentech (Evry, France) under authorization by the ‘Direction Départementale de la Protection des Populations, Ministère de l’Agriculture et de l’Alimentation’, France and in accordance with protocols approved by Xentech along with AstraZeneca’s ‘Platform for Animal Research Tracking aNd External Relationships’ (PARTNER) group. Male athymic nude -Foxn1^nu^ mice aged 6 to 9 weeks were purchased from ENVIGO, France. Animals were housed at density of 6 animals per cage in individual vented cages enriched with sterilized dust free bedding cobs. Animals were identified via unique ear punch numbering system. Animals were acclimatized for a week before entering studies. Housing room temperature was 24 ± 2 °C with humidity at 55 ± 15% with a 14 hour light, 10 hour dark cycle. Animals were fed ad libitum with irradiated rodent diet and drinking water. Xenografts were established by subcutaneous surgical implantation of ~40mm^3^ tumour fragment into the interscapular region under anaesthesia (Ketamine/Xylazine). Tumours were allowed to reach 0.0625-0.196cm^3^ before being randomly assigned into treatment groups.

C4-2 and VcaP studies were performed under contract with Crown Bioscience Inc. (Beijing, China) at AAALAC accredited facilities in accordance with protocols approved by Crown Bioscience along with AstraZeneca’s PARTNER group. C4-2 studies were carried out in male NCG mice aged 6-8 weeks purchased from GemPharmatech Co. Ltd. VcaP studies were carried out in male CB17/SCID mice aged 6-8 weeks purchased from Beijing Vital River Laboratory Animal Technology Co. Ltd. Animals were housed at a density of upto 5 animals per cage in individual vented case enriched with irradiated cardboard tubes, tissue paper and house. Animals were identified via unique ear tag. Animals were acclimatized for a week before entering studies. Housing room temperature was 23 ± 3 °C with humidity at 55 ± 15% with a 12 hour light, 12 hour dark cycle. Animals were fed ad libitum with irradiated rodent diet and drinking water. Tumour cells were implanted subcutaneously into the right flank region, with 5x10^6^ cells per mouse in 0.1ml of equal parts PBS/ Matrigel used for C4-2 implants and 1x10^7^ cells per mouse in 0.1ml of equal parts PBS/Matrigel used in VcaP implants. Mice with tumours ranging 150-200mm3 were randomly assigned into treatment groups.

22RV1 studies was performed under contract with Charles River Laboratories (Freiburg, Germany) at AAALAC accredited facilities in accordance with protocols approved by Charles River Laboratories along with AstraZeneca’s PARTNER group. Male NMRI nu/nu mice (NMRI-Foxn1^nu^) aged 4-6 weeks were purchased from Charles River (Sulzfeld, Germany). Animals were housed at a density of upto 5 animals per cage in individual vented case enriched with aspen wood chips and nesting material. Animals were identified via unique RFID chip. Animals were acclimatized for a week before entering studies. Housing room temperature was 25 ± 1 °C with humidity at 55 ± 15% with a 14 hour light, 10 hour dark cycle. Animals were fed ad libitum with irradiated rodent diet and drinking water. Tumour cells were subcutaneously implanted with 1x10^7^ cells per mouse in 0.2ml of equal parts PBS – Matrigel. Mice with tumours ranging 150-200mm3 were randomly assigned into treatment groups.

CTG-2428 studies were performed under contract with Champions Oncology (Rockville, Maryland, USA) at AAALAC accredited facilities in accordance with protocols approved by Champions Oncology’s IACUC along with AstraZeneca’s PARTNER group. Male NOG mice aged 6-8 weeks were purchased from Taconic Bioscience (NY, USA). Animals were housed at a density of upto 5 animals per cage in individual vented case enriched with irradiated corncob bedding and nesting enrichment sheets. Animals were identified via unique RFID chip. Animals were acclimatized for 3 days before entering studies. Housing room temperature was 21.5 ± 1.5 °C with humidity at 50 ± 20% with a 14 hour light, 10 hour dark cycle. Animals were fed ad libitum with irradiated rodent diet and drinking water. Tumour fragments were implanted subcutaneously tumour fragment. Mice with tumours ranging 150-300mm3 were assigned into treatment groups.

For efficacy studies (PAC120, HID28, VcaP, CTG2428 and 22Rv1 n=10 animals per arm, dosing for 4 weeks, C4-2 n= 5 vehicle, capivasertib monotherapy and docetaxel monotherapy, n=10 capivasertib and docetaxel combination) animals were dosed with; 100mg/kg (PAC120, HID28, VcaP, CTG2428 and C4-2) or 130mg/kg (22RV1) PO BID capivasertib (4 days on 3 days off, starting the day after docetaxel dosing, in 10% DMSO/25% Kleptose, pH5, 10ml/kg); 5mg/kg IV Docetaxel (up to once weekly, starting the day before capivasertib. PAC120 and HID28 dosed weeks 1-4; 22RV1 and VcaP dosed weeks 1-3; CTG-2428 dosed weeks 1 and week 3; C4-2 dosed week 1. Docetaxel dosed in Physiological Saline, 10ml/kg); a combination of capivasertib and Docetaxel (as above); or treated with equivalent vehicle controls. Dosing schedules for each model are highlighted in **Figure 1**.

Across all models, animals observed to have >15% bodyweight loss (BWL) stopped treatment, if BWL >15% for 72 hours or if BWL>20%, animals were removed from study. Tumours were measured by caliper to record tumour width and tumour length, from which presented volume was calculated as; Volume (/cm3) = (π x (Maximum measure (Length or Width)) x (Minimum measure (Length or Width)) x (Minimum measure (Length or Width)))/6000. Percentage change from initial tumour volume was calculated as; % tumor volume change = (‘Final volume’ – ‘Initial volume’)/’Initial volume) x 100. Relative Tumour Volume (RTV) on day ‘X’was calculated as; RTV = (Tumour volume on day X)/(Tumour volume on day 0). Percentage Tumour Growth Inhibition (TGI) on day ‘X’ was calculated as; TGI = (((Vehicle RTV day X) – (Treatment group RTV day X))/((Vehicle RTV on day X) – (Vehicle RTV on day 0))) x 100. When TGI >100%, percentage regression was calculated as; Regression = (RTV on day 0 – RTV on day X) x 100. Final animal numbers used to calculate TGI and Regression are highlighted in **Figure 1 G, H**.

**Cell cycle analysis**

Cells were seeded into T25 flasks at 500,000 to 750,000 cells per flask depending on the cell line in 5mls of culture media and the cells allowed to attach overnight. The cells were treated with DMSO control, monotherapy and the combination as indicated. To determine cells in S phase EdU pulse labelling and staining was carried out using a “Click-iT Plus EdU Alexa Fluor 647 Flow cytometry assay Kit” (Invitrogen # C10634). The manufacturers protocol was followed to prepare the kit reagents. One hour prior to harvesting cells 5µl of 10mM EdU solution was added per 5ml media in the flask and the cells incubated for a further hour. Cells were harvested, washed with PBS and pelleted prior to being fixed by resuspension in 2mls of ice cold 70% ethanol. The cell number for each sample was counted using Trypan Blue stain to determine the live cell number. The cells were washed in blocking buffer (1% BSA/PBS) and pelleted at 300g for 5 minutes at 4°C. The supernatant was carefully removed, and the cells resuspended in 200µl of blocking buffer and transferred to a 96 well plate. The cells were pelleted at 1000rpm for 5 minutes the supernatant removed, and the cells resuspended in 100µl of Click-iT permeabilization and wash reagent and incubated for 15 minutes at RT. The samples were pelleted at 1000rpm for 5 minutes the supernatant removed, and the cells resuspended in 100µl of Click-iT Plus reaction cocktail and incubated for 30 minutes at RT in the dark. The cells were pelleted at 1000rpm for 5 minutes and resuspended in 200µl of Click-iT permeabilization and wash reagent. The cells were pelleted the supernatant removed and the cells resuspended in 100µl of Phospho-Histone H3(S10) (D2C8) XP Rabbit mAb (Alexa Fluor 488 Conjugate) (1 in 50 dilution in 1% BSA/PBS) (Cell Signaling Technology #3465). The samples were incubated with antibody for 45 minutes at RT in the dark. 100µl per well of PBS was added and the cells pelleted at 1000rpm for 5 minutes. The supernatant was carefully aspirated from each well and the cells resuspended in 200µl per well of FxCycle Violet working solution (1 in 1000 in 1% BSA/PBS). The samples were incubated at RT for at least 30 minutes prior to FACS analysis. Fluorescence was captured on a FACS Celesta Flow Cytometer (BD Biosciences) and cell cycle analysis carried out using FlowJo v8.

***In vitro* senescence assay**

The Mammalian β-galactosidase assay kit (Thermoscientific cat #75707) was used to detect beta-galactosidase activity in lysates prepared from the adherent cells remaining following treatment of DMSO and docetaxel pre-treated cells further treated with DMSO control or capivasertib. The protocol was followed as indicated in the manufacturers protocol. Briefly prostate cells were seeded across 4 wells of a 24 well plate at 50,000 – 200,000 cells per well depending on the cell line. The following day for each cell line 2 wells were treated with DMSO control and 2 wells with 10nM docetaxel and the plates incubated overnight. The following day suspension cells were removed, and the adherent cells washed twice with PBS. The adherent cells were treated with DMSO control or capivasertib for a further 24 hours. The following day the cells were washed and lysed in 100µl of MPER protein extraction reagent per well. The plate was incubated at room temperature for 5 minutes to allow lysis. 50µl of lysate was transferred to a 96 well plate across two replicate wells per lysate and 50µl per well of β-galactosidase assay reagent added per well. The plate was covered with an adhesive lid and incubated at 37°C for 30 minutes. The OD 405nm was measured on the Envision. The average and standard deviation data was plotted in PRISM.

**Supplementary Results Cell Cycle Analysis**

**Detailed cell cycle characterisation of suspension and docetaxel-persister prostate cancer cells treated with 10 and 100nM docetaxel for 24 hours.**

Consistent with previous reports for mitotic spindle inhibitors, cells showed variable sensitivity to the effects of low and high concentrations of docetaxel on cell cycle ^1^. Across all cell lines, treatment with 100nM docetaxel increased the number of cells in subG1 and G2 of the suspension cell population with increased phospho-histone H3 positivity (mitotic index (MI)) indicating cell death and G2/M mitotic arrest in these cells. Similarly, in the suspension cell population, treatment with 10nM docetaxel increased the number of cells in subG1, however an increase in G2 with an associated increase in MI was only observed in VCaP and LNCaP cells with an increase in MI in PC3 cells. In 22RV1 and PC346Flu1 suspension cell fractions, the number of cells in G2/M was not altered with 10nM docetaxel treatment. Finally, the extent of cell cycle arrest induced by 10 and 100nM docetaxel in the suspension cell population varied across cell lines irrespective of PTEN status with highest levels observed in LNCaP cells (96-98%) and lowest in PC346Flu1 cells (0.4-2%) (**Supplementary Table 1**).

In the adherent cell population, the effects of docetaxel monotherapy on cell cycle were variable and did not associate with PTEN status **(Supplementary Table 1)**. The adherent cell populations pre-treated with 100nM docetaxel were mainly in G2 with increased phospho-histone H3 positivity (MI). In VCaP and PC346Flu1 the increase in cells in G2 with associated increased MI was less with the larger fraction remaining in G1 and active S phase. In the adherent cell fraction, 10nM docetaxel treatment resulted in an increase in subG1 across all cell lines, with a decrease in G1 and an increase in G2, although quite variable across cell lines, and the percentage of G2 cells staining positive for phospho-histone H3 (MI) was less than that observed in adherent cells following treatment with 100nM docetaxel.

**Detailed cell cycle characterisation of short-term-docetaxel-persister cells treated with docetaxel and capivasertib monotherapy and the combination**

The impact of the monotherapy and combination treatment on cell cycle in the docetaxel-persister cells were analysed by flow cytometry **(Supplementary Figure 3 A, B)**. In LNCaP, PC3 and PC346Flu1 cells that are more sensitive to AKT inhibition (data not shown) capivasertib monotherapy treatment increased the percent of cells in sub-G1/G1 consistent with previous reports, ^2^ **(Supplementary Figure 3B)**. The combination treatment reduced the total number of persister cells relative to docetaxel monotherapy treatment (**Supplementary Figure 3A**) however, the percentage of cells in each cell cycle phase was similar (**Supplementary Figure 3B**). Western blot analysis for expression of proteins associated with cell cycle control showed that in PTEN null (C4-2, LNCaP, and PC3) and PTEN WT (22RV1 and VCaP) cells reduced levels of cyclin B1 (indicative of mitotic exit) and reduced cyclin D1 and/or phospho-Rb (S807/811) levels (indicative of G1 arrest) after combination treatment (**Supplementary Figure 4A**). Docetaxel induced total p21 levels in p53 functional, (C4-2, LNCaP, PC345Flu1 and 22RV1) and p53 non-functional (PC3 and VCaP) cells (**Supplementary Figure 4A**). In contrast, capivasertib treatment alone or in combination, decreased p21 levels and induced p27 in C4-2, LNCaP and PC346Flu1 cells, consistent with p27 being a direct target of AKT^3,4^, (**Supplementary Figure 4A**). Reduced p21 and increased p27 expression has previously been associated with a senescence-like phenotype following PI3K/AKT pathway inhibition^5^. However, induction of the senescence marker beta-galactosidase ^6^ was not observed in C4-2, LNCaP or PC346Flu1 docetaxel-persister cells treated for 24 hours with capivasertib (**Supplementary Figure 4B**).

**Supplementary Figure Legends**

**Supplementary Figure 1.** Subcutaneous xenograft and PDX models of PTEN null (**A, B, C**) and PTEN WT (**D, E, F**) prostate cancer were dosed for up to 28 days. Each bar represents the % change in tumour volume from initial tumour volume for individual tumours, on the final day animals were on study.

**Supplementary Figure 2** (**A**) Western blot analysis of indicated PI3K/AKT and apoptotic pathway markers in lysates prepared from suspension and adhered LNCaP and VCaP cells after 24 hour treatment with 10 and 100nM docetaxel and DMSO control. Cleaved and full-length bands were quantified using GeneTools (Syngene) and are shown for each sample as fold change relative to DMSO control. Data shown is representative of two replicate experiments. (**B**) Western blot analysis of cleaved and full-length PARP in lysates prepared from suspension and adhered PC3, PC346Flu1 and 22RV1 cells after 24 hour treatment with 10 and 100nM docetaxel and DMSO control. Cleaved and full-length bands were quantified using GeneTools (Syngene) and are shown for each sample as fold change relative to DMSO control.

**Supplementary Figure 3**. (**A**) Plots showing the number of adherent cells remaining, after each indicated treatment, plotted relative to DMSO control. Data is an average of two independent experiments. Error bars represent standard deviation. (**B**) Plots showing Flow cytometry cell cycle profiles of adherent cells following treatment with DMSO control, 10nM docetaxel, 750nM capivasertib and a combination. Data is plotted as percentage of total live cells in each phase and is shown as average of two independent experiments with error bars representing standard deviation.

**Supplementary Figure 4.** (**A**) Western blot profile of indicated cell cycle control markers in lysates from adherent PTEN null and PTEN WT cells pre-treated with 10nM docetaxel and DMSO control for 24 hours followed by wash off and subsequent treatment with 750nM capivasertib and DMSO control for 24 hours. (**B**) Plot showing the effect of 10nM docetaxel and 750nM capivasertib monotherapy and combination on beta-galactosidase activity in lysates from adherent C4-2, LNCaP and PC346Flu1 cells pre-treated with 10nM docetaxel and DMSO control for 24 hours followed by wash off and subsequent treatment with 750nM capivasertib and DMSO control for 24 hours. Error bars represent standard deviation across two replicate wells. Data is representative of two identical experiments.

**Supplementary Figure 5.** (**A**) Western blot analysis and (**B**) quantification of pGSK3b(S9) and cleaved and full-length PARP levels in lysates from adherent cells treated with 10nM docetaxel and DMSO control for 24 hours followed by wash off and subsequent treatment with 750 and 2000nM capivasertib and DMSO control for 24 hours. Cleaved and full-length PARP and pGSK3b(S9) bands were quantified using GeneTools (Syngene) and data plotted in PRISM v9.

**Supplementary Figure 6.** (**A**) Schematic of the assay procedure. (**B**) Western blot analysis of pTau(T209) in lysates from 22RV1 cells pre-treated 1, 3, 10 and 100nM docetaxel and DMSO control for 24 hours followed by wash off and subsequent treatment with 1000nM AZD2858 and 2000nM capivasertib alone and in combination for 24 hours. (**C**) Western blot analysis of indicated markers in lysates from C4-2, PC346Flu1 and 22RV1 cells pre-treated 1, 3, 10 and 100nM docetaxel and DMSO control for 24 hours followed by wash off and subsequent treatment with 1000nM AZD2858 and 2000nM capivasertib alone and in combination for 24 hours. Data is representative of two similar experiments.

**Supplementary References**

1. Chen JG, Horwitz SB. Differential mitotic responses to microtubule-stabilizing and -destabilizing drugs. *Cancer Res*. Apr 1 2002;62(7):1935-8.

2. Thomas C, Lamoureux F, Crafter C, et al. Synergistic targeting of PI3K/AKT pathway and androgen receptor axis significantly delays castration-resistant prostate cancer progression in vivo. *Mol Cancer Ther*. Nov 2013;12(11):2342-55. doi:10.1158/1535-7163.MCT-13-0032

3. Rassidakis GZ, Feretzaki M, Atwell C, et al. Inhibition of Akt increases p27Kip1 levels and induces cell cycle arrest in anaplastic large cell lymphoma. *Blood*. Jan 15 2005;105(2):827-9. doi:10.1182/blood-2004-06-2125

4. Chen R, He F, He H, York JP, Liu W, Xia X. Phosphorylation of P27 by AKT is required for inhibition of cell cycle progression in cholangiocarcinoma. *Dig Liver Dis*. May 2018;50(5):501-506. doi:10.1016/j.dld.2017.12.021

5. Collado M, Medema RH, Garcia-Cao I, et al. Inhibition of the phosphoinositide 3-kinase pathway induces a senescence-like arrest mediated by p27Kip1. *J Biol Chem*. Jul 21 2000;275(29):21960-8. doi:10.1074/jbc.M000759200

6. Gonzalez-Gualda E, Baker AG, Fruk L, Munoz-Espin D. A guide to assessing cellular senescence in vitro and in vivo. *FEBS J*. Jan 2021;288(1):56-80. doi:10.1111/febs.15570
