# Supplementary material for: Capivasertib combines with docetaxel to enhance anti-tumour activity through inhibition of AKT-mediated survival mechanisms in prostate cancer
Source: Br J Cancer. 2024 Feb 23;130(8):1377–87. doi: 10.1038/s41416-024-02614-w (PMC11014923; doi:10.1038/s41416-024-02614-w)

Supplementary Figure 1

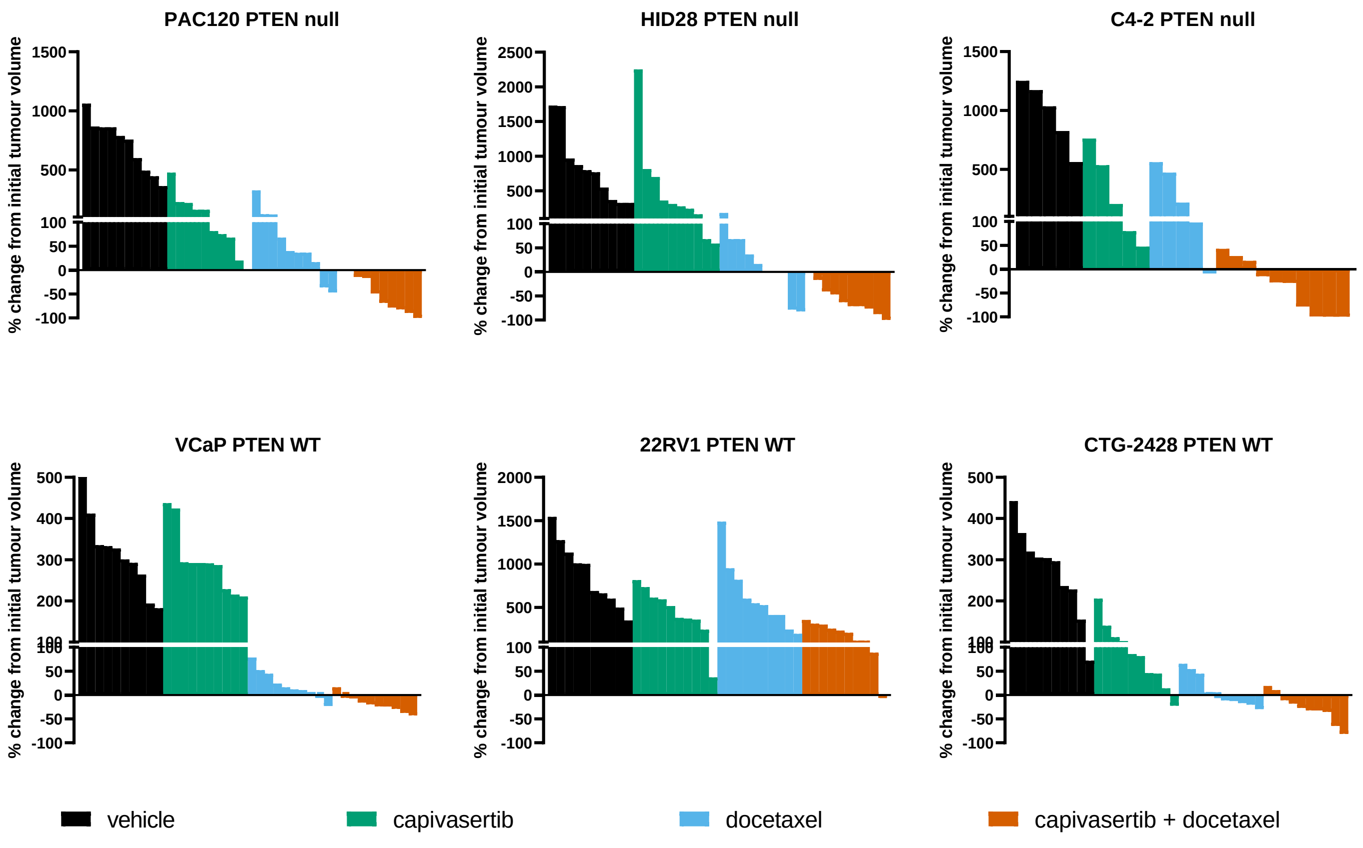

Supplementary Figure 2

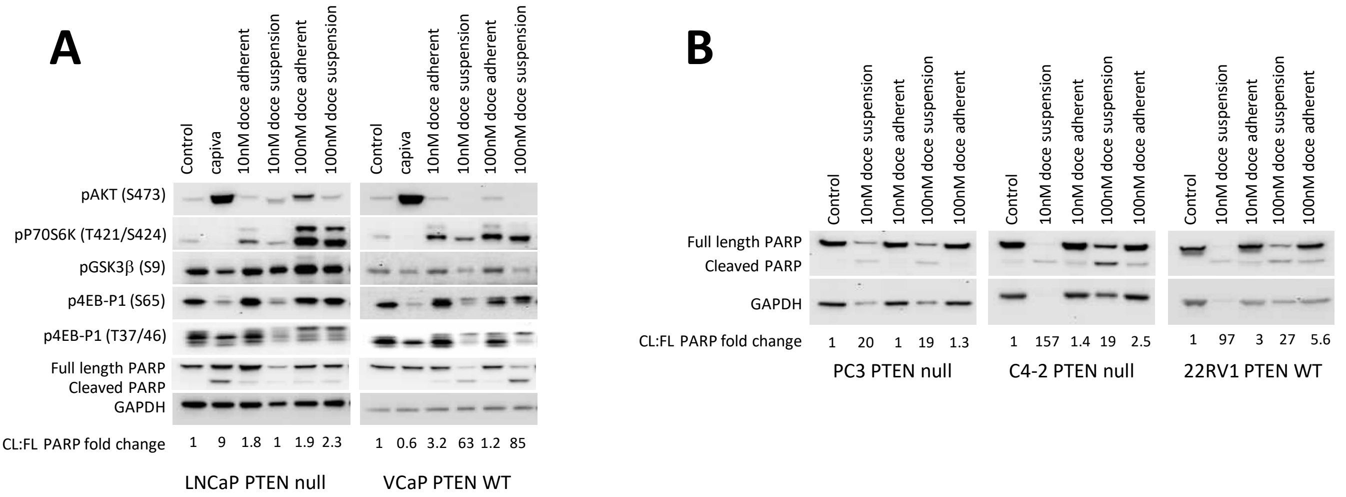

Supplementary Figure 3

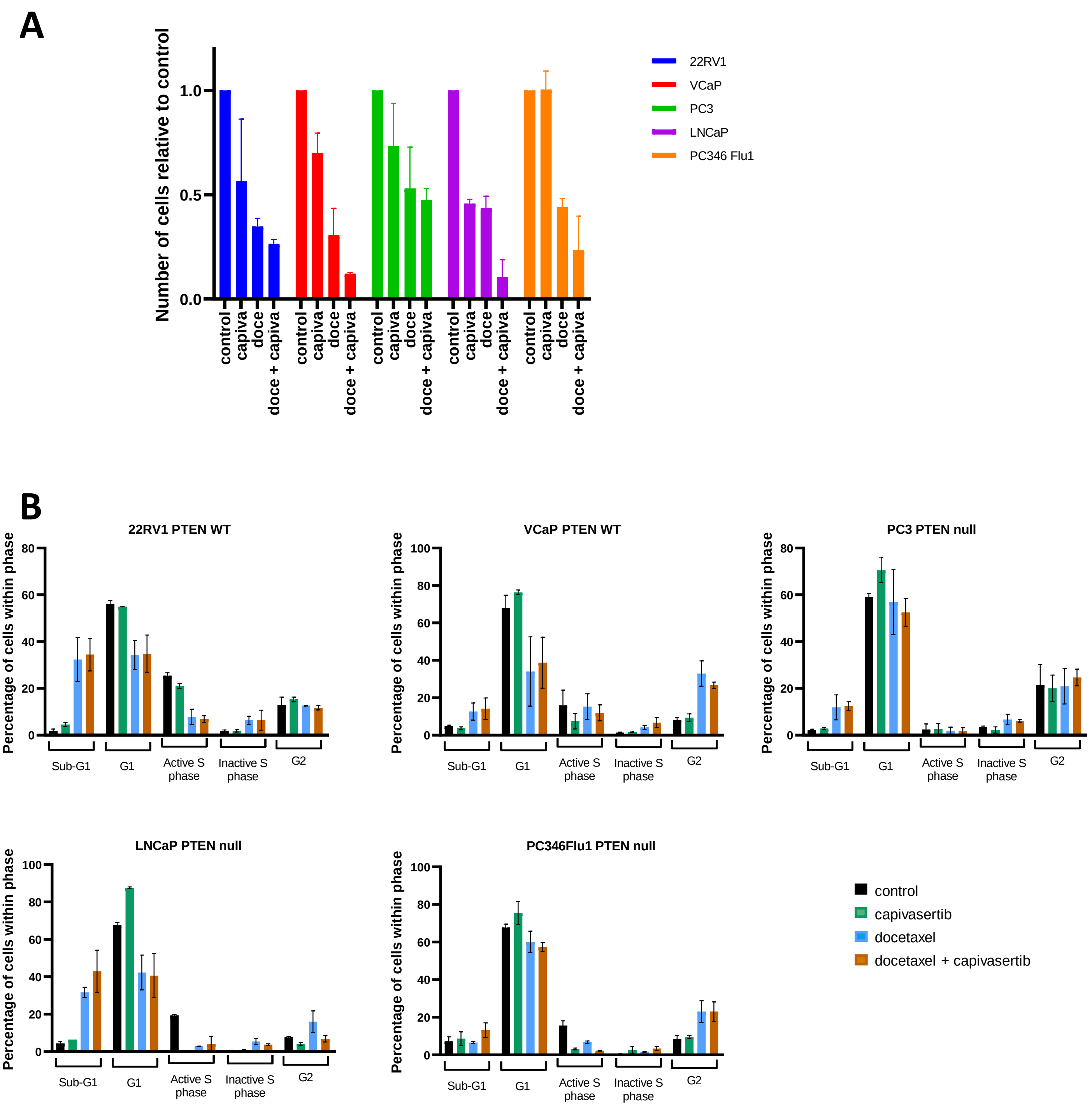

Supplementary Figure 4

A

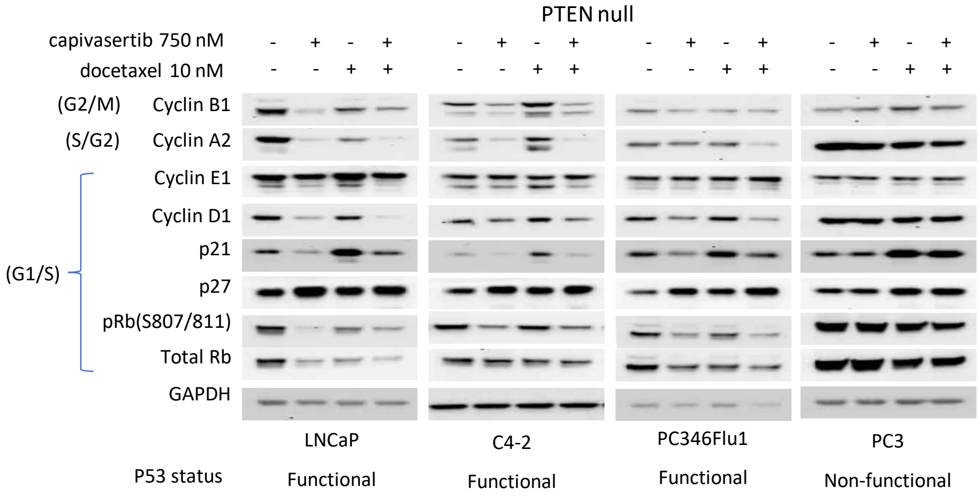

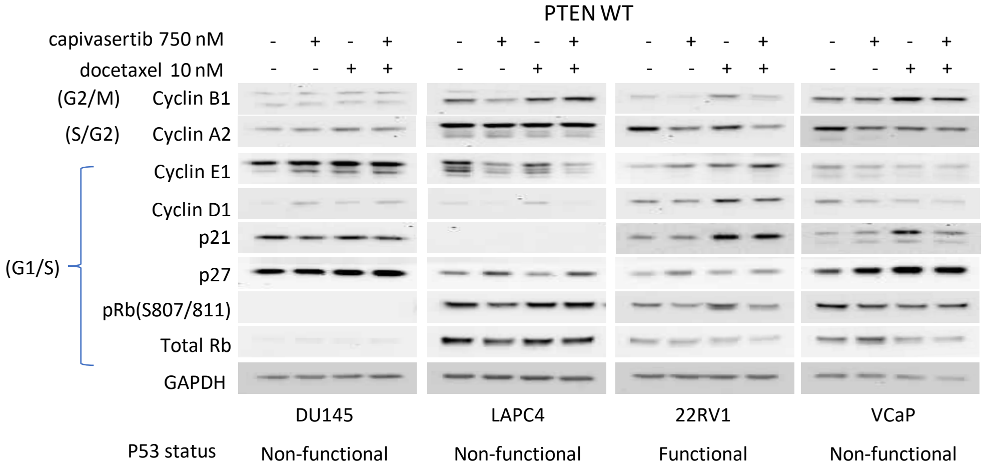

B

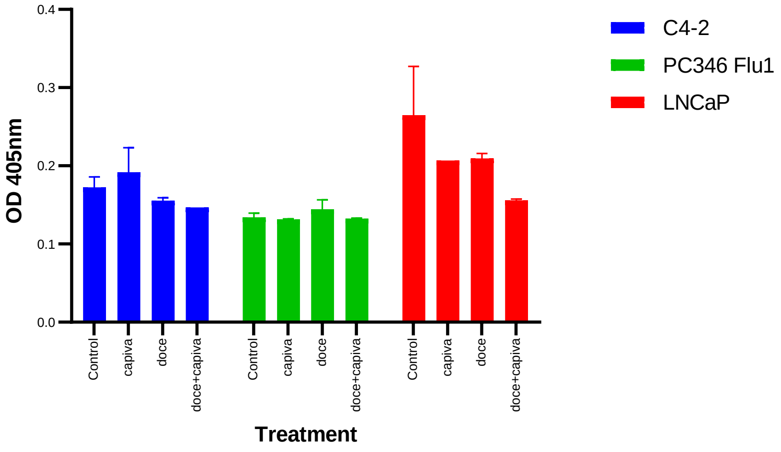

Supplementary Figure 5

A

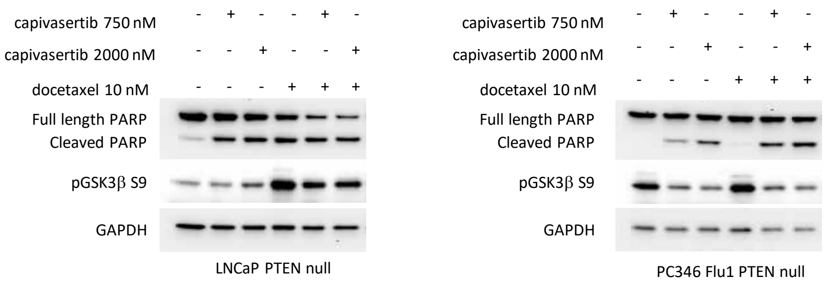

B

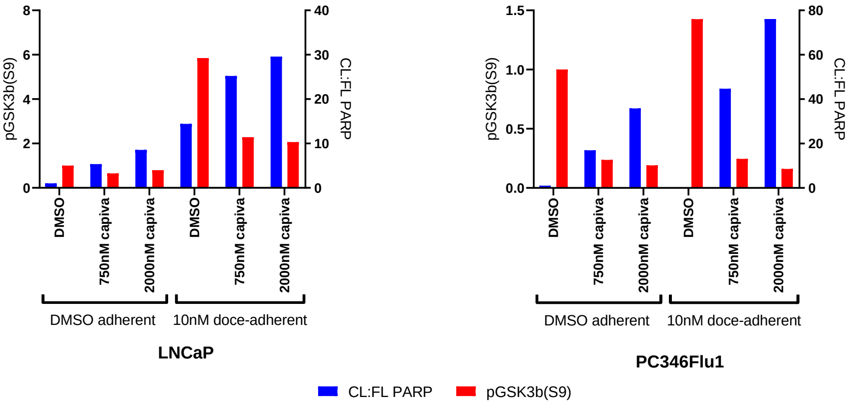

Supplementary Figure 6

A

Seed cells → 24 hours → Treat with 1, 3, 10 and 100nM docetaxel or DMSO control → 24 hours → Wash off docetaxel and treat cells with AZD2858 and capivasertib alone or combination → 24 hours → Wash and harvest cells for analysis

B

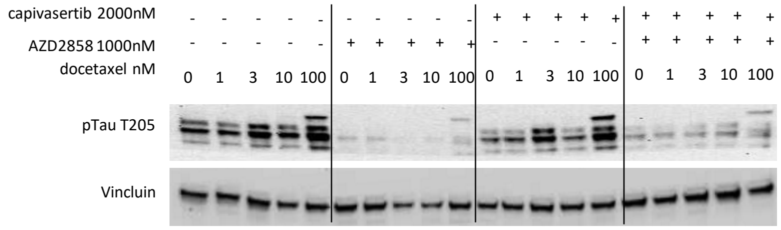

C

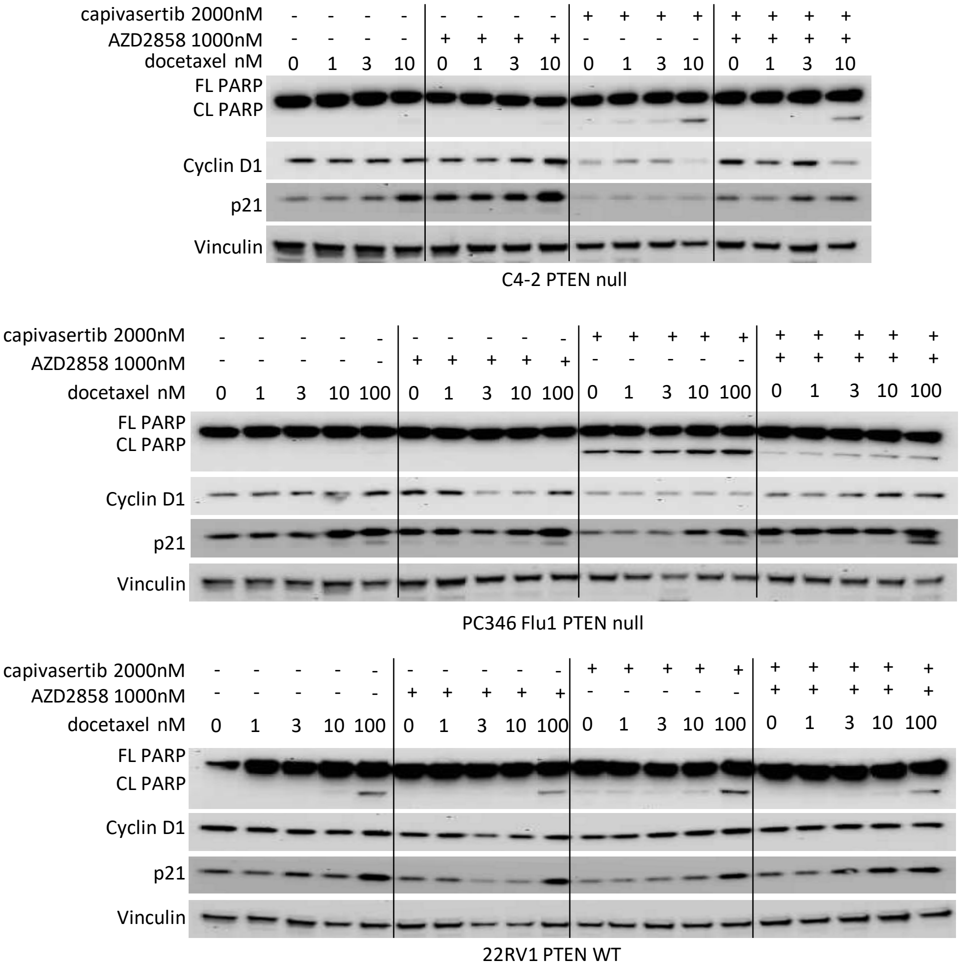

Supplement: Supplementary file 2 — Supplementary Figures [file 41416_2024_2614_MOESM2_ESM.pdf]
